# An eight-gene prognostic signature and its immune associations in gastric cancer: integrated transcriptomic and clinical analyses

**DOI:** 10.3389/fonc.2026.1930319

**Published:** 2026-10-22

**Authors:** Dong Wen, Lili Mi, Yan Zhang, Yaming Ji, Baoling Yang, Fei Yin

**Affiliations:** 1 Department of Gastroenterology, The Fourth Hospital of Hebei Medical University, Shijiazhuang, Hebei, China; 2 Department of Gastroenterology, Baoding No. 1 Central Hospital, Baoding, Hebei, China; 3 Department of Pathology, Baoding No. 1 Central Hospital, Baoding, Hebei, China

**Keywords:** gastric cancer, Helicobacter pylori, immunotherapy, prognostic biomarker, tumor immune microenvironment

## Abstract

**Background:**

Gastric cancer (GC) remains a major cause of cancer-related mortality worldwide. *Helicobacter pylori* (*H. pylori*) is a major etiological factor in gastric carcinogenesis and has been associated with alterations in the tumor immune microenvironment. However, how *H. pylori*-related transcriptional features intersect with the broader molecular and immune heterogeneity of established gastric cancer remains incompletely characterized.

**Methods:**

Single-cell RNA-sequencing data (GSE234129) were used for exploratory comparison according to *H. pylori* status and to define an initial candidate gene set. An eight-gene prognostic signature was subsequently developed in TCGA-STAD and evaluated in independent gastric cancer cohorts. Model-development optimism was assessed using 1, 000 full-pipeline bootstrap resamples. Immune features were evaluated using CIBERSORT, ESTIMATE, TIDE, and immunophenoscore, while observed anti-PD-1 response was explored in the disease-matched PRJEB25780 cohort. RGS1 was further assessed in an independent immunohistochemical cohort.

**Results:**

Exploratory single-cell analyses suggested differences in immune-cell composition, T-cell states, and antigen-presentation-related pathways according to *H. pylori* status. The eight-gene prognostic signature stratified overall survival in the TCGA-STAD cohort and two independent GEO cohorts. After full-pipeline bootstrap correction, model discrimination was modest (corrected C-index = 0.575). In TCGA-STAD, addition of the eight-gene score to a clinicopathological model improved prognostic discrimination. High-risk tumors were characterized by distinct stromal, genomic, and immune features, including higher stromal activity, lower tumor mutational burden, and differences in computational immune-response-related profiles. At the tissue level, RGS1 expression was associated with survival and selected immune-cell features. In the IHC cohort, high RGS1 expression remained independently associated with poorer overall survival (OS) after multivariable adjustment (HR = 2.36, 95% CI 1.36-4.10, *p* = 0.002) and was accompanied by lower CD8-positive and higher CD163-positive and FOXP3-positive cell densities. In the independent *H. pylori*-annotated GSE62254 cohort, the five non-ribosomal components showed nominal gene-set-level enrichment in *H. pylori*-positive tumors (CAMERA P = 0.044).

**Conclusions:**

This study developed an eight-gene survival-associated prognostic signature and characterized its immune associations in gastric cancer. *H. pylori*-related findings were exploratory and did not establish *H. pylori* specificity of the final model. Further prospective validation is required given the modest optimism-corrected performance.

## 1 Introduction

Gastric cancer (GC) remains a major cause of cancer-related mortality worldwide, accounting for approximately 4.9% of all cancer diagnoses and 6.8% of cancer deaths globally (1). In China, GC ranks third in cancer mortality (2). Despite advances in surgical and systemic therapies, recurrence and metastasis continue to contribute substantially to poor outcomes, particularly in patients presenting with advanced disease and marked biological heterogeneity (3, 4). Recent phase III trials, including CheckMate-649, ORIENT-16, and KEYNOTE-811, have demonstrated clinically meaningful survival benefits from first-line immunotherapy in selected patients with advanced GC. Nevertheless, primary and acquired resistance remain common, highlighting the need for biomarkers that can improve risk stratification and further characterize clinically relevant tumor immune heterogeneity (5–8).

Recent systematic reviews indicate that the global prevalence of *H. pylori* infection has declined but remains above 40%, with substantial geographic variation (9). In China, the pooled prevalence during 2014–2023 was estimated at approximately 42.8% (10). *H. pylori* is estimated to account for nearly 90% of non-cardia gastric cancers worldwide, although only 1-3% of infected individuals ultimately develop GC (11–13). *H. pylori* infection is associated with increased tumor expression of programmed death-ligand 1 (PD-L1), suggesting that infection status may be associated with differential immunotherapy-related immune features (14–16). Some studies have further associated *H. pylori* positivity with an inflamed or “hot” tumor immune phenotype that may favor immune checkpoint inhibitor (ICI) activity (17), whereas others have reported shorter progression-free survival and overall survival among *H. pylori*-positive patients treated with ICIs (18). These apparently conflicting findings underscore the complex and incompletely understood relationship between *H. pylori* status, tumor immune features, clinical outcomes, and treatment response in established GC.

Although *H. pylori* is a well-established etiological factor in gastric carcinogenesis, its clinical and immunological relevance after malignant transformation remains less well defined. Previous studies have largely focused on *H. pylori*-induced chronic inflammation, gastric mucosal injury, and the progression of precancerous lesions (19). In established GC, however, whether transcriptional and immune features associated with *H. pylori* status persist and carry prognostic relevance remains incompletely characterized. Components of the tumor immune microenvironment, including tumor-infiltrating lymphocytes, regulatory T cells, tumor-associated macrophages, and PD-L1 expression, have been associated with immune regulation, tumor progression, prognosis, and treatment response in GC (20–24). These observations raise the possibility that *H. pylori* status may be associated with clinically relevant tumor heterogeneity beyond its established role in gastric carcinogenesis.

Numerous gene expression-based prognostic signatures have been proposed for GC, reflecting the substantial molecular heterogeneity of this disease (25). Large-scale molecular studies have also identified distinct genomic and transcriptomic subtypes of GC with different biological characteristics and clinical outcomes (26, 27). However, most have been derived primarily from tumor transcriptomic characteristics without explicitly incorporating etiological context. *H. pylori*-related transcriptional differences may provide one exploratory entry point for identifying molecular features relevant to the heterogeneity of established gastric cancer. Subsequent survival-based modelling and immune characterization may help determine whether genes identified in this exploratory context are associated with clinically relevant tumor phenotypes.

Accordingly, we integrated exploratory single-cell RNA sequencing, bulk transcriptomic datasets, clinical outcome data from multiple cohorts, and tissue-level analyses to investigate molecular and immune heterogeneity in gastric cancer. Exploratory single-cell comparisons according to *H. pylori* status were first used to define a candidate gene set, from which an eight-gene survival-associated prognostic signature was subsequently developed and evaluated. This integrative framework was designed to characterize the prognostic and immune associations of the resulting multigene signature while retaining *H. pylori*-related analyses as exploratory biological context.

## 2 Materials and methods

### 2.1 Data collection

RNA-sequencing data and clinical information for the TCGA-STAD cohort (n = 383) were obtained from The Cancer Genome Atlas. Two independent gastric cancer cohorts, GSE26899 and GSE15459, were obtained from the Gene Expression Omnibus (GEO). The scRNA-seq dataset GSE234129 was used for exploratory analysis of *H. pylori*-associated transcriptional and immune features. PRJEB25780 was used as a disease-matched gastric cancer anti-PD-1 cohort for exploratory treatment-response analyses. The IMvigor210 urothelial carcinoma cohort was included as an exploratory cross-tumor immunotherapy dataset. GSE62254 was additionally analyzed as an exploratory *H. pylori*-annotated gastric cancer cohort to assess whether components of the final signature retained transcriptional associations with *H. pylori* status. **Figure 1** summarizes the study design.

**Figure 1:**
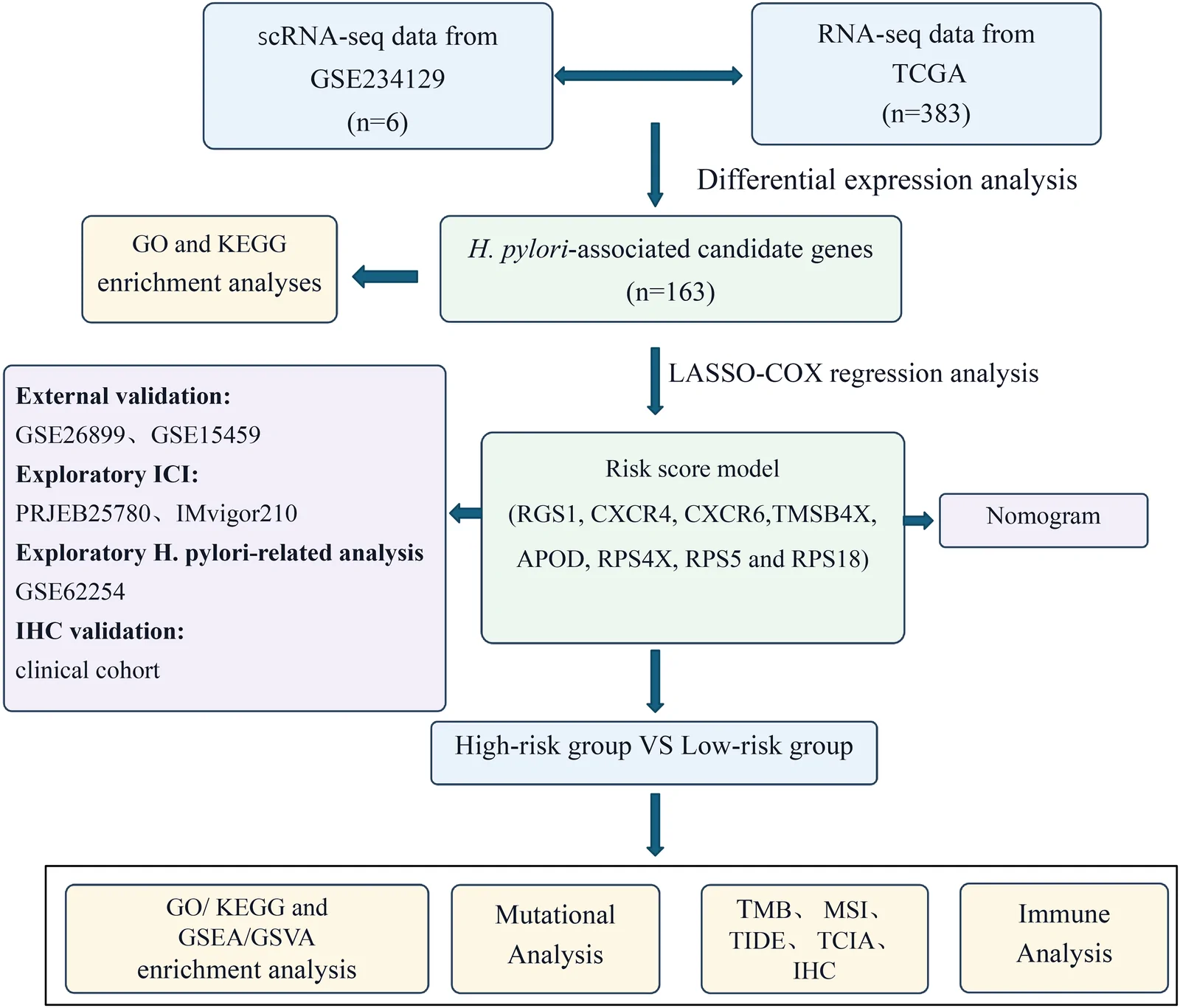
The flowchart for this study.

### 2.2 Exploratory candidate gene screening according to *H. pylori* status and pseudobulk analysis

The GSE234129 dataset was processed using Seurat. Genes detected in at least three cells were retained; cells with mitochondrial transcript content ≤15% and hemoglobin transcript content < 5% were included, yielding 5,109 high-quality cells. Principal component analysis (PCA) was performed using the top 2,000 highly variable genes. Cell clusters were identified using FindClusters and FindNeighbors functions and annotated according to established marker genes (28). At the cell level, genes showing expression restricted to the *H. pylori*-positive group, a detection rate ≥25%, and an adjusted P value <0.05 were retained as exploratory candidate genes. These candidate genes were subsequently intersected with genes available in TCGA-STAD for downstream prognostic modelling. To address potential cellular pseudoreplication, raw T-cell counts were additionally aggregated at the patient level, yielding three *H. pylori*-positive and three *H. pylori*-negative pseudobulk samples. Patients with at least 20 T cells were retained. Differential expression was analyzed using edgeR with trimmed mean of M values (TMM) normalization and a quasi-likelihood framework, after lowly expressed genes were removed using filterByExpr. Genes with |log2FC| ≥ 1 and false discovery rate (FDR) < 0.05 were considered statistically significant, whereas genes with nominal *p* < 0.05 and |log2FC| ≥ 1 were considered exploratory signals. The patient-level pseudobulk analysis was used as a sensitivity analysis to assess the robustness of the cell-level findings.

### 2.3 Eight-gene prognostic signature development and external evaluation

Candidate genes were first evaluated using univariable Cox proportional hazards regression in the TCGA-STAD cohort. To avoid prematurely excluding variables with potential prognostic relevance, genes with *p* < 0.20 were carried forward for model development. These candidate genes were entered into LASSO Cox regression with 10-fold cross-validation, and genes with non-zero coefficients at the minimum cross-validated penalty parameter (λmin) were retained. These genes were subsequently entered into a multivariable Cox model followed by bidirectional AIC-based stepwise selection to derive the final eight-gene prognostic signature. The risk score was calculated as a weighted linear combination of gene-expression values using the corresponding Cox regression coefficients. Patients were classified into high- and low-risk groups according to the median risk score. Kaplan-Meier (KM) survival curves, time-dependent ROC analysis, and risk-distribution plots were used to characterize the prognostic performance of the signature. The signature was further evaluated in the two independent gastric cancer cohorts, GSE26899 and GSE15459.

### 2.4 Internal validation and comparative prognostic analyses

To assess model-development optimism, the complete prognostic modelling pipeline, including univariable Cox screening, LASSO Cox regression, multivariable Cox modelling, and bidirectional AIC-based stepwise selection, was repeated in 1,000 bootstrap resamples, with optimism-corrected Harrell’s C-index and 1-, 3-, and 5-year time-dependent AUCs calculated. The incremental prognostic value of the eight-gene score was evaluated by comparing a clinicopathological model (age, sex, and pathological stage), the eight-gene model, and their combined model; a separate 1,000-resample fixed-score bootstrap was used to assess the stability of model discrimination. The eight-gene score was also benchmarked against a previously published 32-gene gastric cancer signature in the same TCGA-STAD patients using the same survival endpoint (29). Among the eight signature genes, RGS1 was additionally evaluated as a single-gene prognostic marker because it was selected for subsequent biological analyses. Its prognostic discrimination was compared with that of the eight-gene signature in the same TCGA-STAD patients using Harrell’s C-index and 1-, 3-, and 5-year time-dependent AUCs. A nested likelihood-ratio test further compared the RGS1-only Cox model with a Cox model containing all eight signature genes. Because three genes in the final signature encoded ribosomal proteins, the complete prognostic modelling pipeline was additionally repeated after exclusion of all RPS/RPL genes. In a complementary fixed-coefficient sensitivity analysis, the coefficients of the five non-ribosomal genes were retained from the original eight-gene model, while the contributions of RPS4X, RPS5, and RPS18 were removed from the risk-score formula without model refitting. In GSE62254, the five non-ribosomal components of the signature were evaluated as an exploratory gene set using CAMERA. Four exploratory gene sets were evaluated, and P values were additionally adjusted using the Benjamini–Hochberg method. In addition, the eight-gene score was calculated in GSE62254 using the fixed TCGA-derived coefficients after gene-wise standardization and was compared between *H. pylori*-positive and *H. pylori*-negative tumors using the Wilcoxon rank-sum test.

### 2.5 Consensus clustering and nomogram

Molecular subtypes in TCGA-STAD were identified using ConsensusClusterPlus. PCA was used to visualize subtype separation. Univariable and multivariable Cox regression were used to evaluate the prognostic associations of clinical covariates. A nomogram integrating risk score, age, and tumor stage was developed using the rms package. Model performance was evaluated by time-dependent ROC analysis, calibration curves, decision curve analysis (DCA), and the concordance index (C-index).

### 2.6 Mutation and pathway enrichment analysis

Somatic mutation data from cBioPortal were analyzed using maftools to characterize the mutation landscape and calculate tumor mutational burden (TMB), whereas MSI information was obtained from the corresponding TCGA annotations. Gene Ontology (GO), KEGG, and Hallmark gene set enrichment analyses were used to assess pathway differences between the high- and low-risk groups. Gene Set Variation Analysis (GSVA) was used to quantify pathway-level activity scores. Hallmark gene sets were obtained from the corresponding gene-set resource accessed in February 2025.

### 2.7 Immune infiltration and exploratory immunotherapy response analysis

Immune cell composition was estimated using CIBERSORT (30), whereas stromal and immune scores were calculated using ESTIMATE (31), with additional immune signatures obtained from TISIDB (32). TIDE and immunophenoscore (IPS) were used to assess computational immunotherapy-related features (33, 34). Associations of RGS1 expression and the eight-gene score with observed anti-PD-1 response were explored in the PRJEB25780 gastric cancer cohort, and the correlation between RGS1 expression and the eight-gene score was assessed using Spearman’s rank correlation. The IMvigor210 urothelial carcinoma cohort was included only as an exploratory cross-tumor analysis and was not considered a disease-matched validation cohort.

### 2.8 Patients and clinical samples

Tumor tissues from 128 patients with GC who underwent surgical resection between October 2020 and May 2023 were collected for immunohistochemical (IHC) analysis. Eligible patients were aged 18–80 years and had histologically confirmed gastric cancer. *H. pylori* status was retrospectively determined from available clinical diagnostic records, including the ^13^C-urea breath test, serological antibody testing, and tissue-based immunohistochemistry. All patients had at least one documented *H. pylori* diagnostic result. Among patients assessed using more than one diagnostic method, all available results were concordant. A sensitivity analysis excluding patients assessed by serology alone was performed to evaluate potential diagnostic-method-related misclassification. Patients who had received preoperative chemotherapy or radiotherapy, had other malignancies, died within 3 months after surgery, had distant metastasis, or had severe hepatic or renal dysfunction were excluded. The study was conducted in accordance with the Declaration of Helsinki and approved by the Ethics Committee of Baoding No. 1 Central Hospital (Ethics approval number k[2024]140). Informed consent was obtained from patients or legal guardians.

### 2.9 Immunohistochemistry

Immunohistochemistry was performed on 128 gastric cancer tissues and 40 adjacent normal tissues using antibodies against RGS1 (rabbit, 1:100; Proteintech), CD163 (mouse, 1:200; Talent Biomedical), CD8 (mouse, 1:200; Talent Biomedical), and FOXP3 (mouse, 1:200; Talent Biomedical). Formalin-fixed, paraffin-embedded tissues were sectioned at 4 μm, deparaffinized, subjected to EDTA-based antigen retrieval, incubated with primary and secondary antibodies, developed with DAB, and counterstained with hematoxylin. Two experienced pathologists independently evaluated the slides while blinded to clinical outcomes. The staining intensity was scored as follows: 0 (negative), 1 (weak), 2 (moderate), and 3 (strong). Staining extent was scored based on percentage of positively stained cells: 0 (0%), 1 (1–25%), 2 (26–50%), 3 (51%–75%), and 4 (76–100%). The final IHC score was calculated by multiplying the intensity and extent scores. For survival analyses, patients were classified into RGS1-high and RGS1-low groups according to the median IHC score.

### 2.10 Statistical analysis

Continuous variables were compared using Student’s t-test or the Wilcoxon rank-sum test, as appropriate, and categorical variables using the chi-square test or Fisher’s exact test. Correlations were assessed using Spearman’s rank correlation. Survival differences were evaluated using Kaplan-Meier analysis and the log-rank test, and Cox proportional hazards regression was used to estimate hazard ratios (HRs) and 95% confidence intervals (CIs). The clinical cohort included 128 patients; however, multivariable Cox regression analyses were based on 126 complete cases because perioperative treatment information was unavailable for two patients. The proportional hazards assumption was assessed using Schoenfeld residuals. For model-comparison analyses, differences in Harrell’s C-index and time-dependent AUC were assessed using paired analyses when the same patients were included in both models. Nested likelihood-ratio testing was used to evaluate incremental model fit where appropriate. All tests were two-sided, with p < 0.05 considered statistically significant unless otherwise specified. Analyses were performed using R version 4.3.3 and GraphPad Prism version 10.1.2.

## 3 Results

### 3.1 Exploratory differences in the GC immune microenvironment according to *H. pylori* status

The GSE234129 single-cell dataset included three *H. pylori*-positive and three *H. pylori*-negative GC patients. After quality control (**Supplementary Figure S1A**), PCA was performed to assess interpatient variation (**Supplementary Figure S1B**), and highly variable genes were identified for downstream dimensionality reduction and clustering (**Supplementary Figure S1C**). Initial clustering identified 17 cell clusters (**Supplementary Figure S1D**), which were subsequently annotated into eight major cell types (**Figure 2A**) according to established marker genes. The cell types included endothelial cells (VWF, PECAM1, CDH5), fibroblasts (PDGFRB, COL1A1, LUM), T cells (CD3D, CD3E), B cells (BANK1, MS4A1, CD79A), monocytes (LYZ, MS4A6A, CD14), NK cells (NKG7, NCR1), plasma cells (MZB1, IGHG1, SDC1), and mast cells (GATA2, KIT, TPSAB1, CPA3) (**Figure 2B**). Visualization according to *H. pylori* status showed differences in the distribution of major cell populations (**Figure 2C**). Cell-type composition varied substantially across individual patients, with T-cell proportions ranging from 35.1% to 84.5%. MDA_Pt9 showed marked T-cell predominance, whereas other patients exhibited variable enrichment of monocytic and plasma-cell populations (**Figure 2D**). Descriptive comparison of cell-type proportions according to *H. pylori* status suggested relatively lower proportions of monocytes, mast cells, and fibroblasts and a higher proportion of T cells in the *H. pylori*-positive samples (**Supplementary Figure S1E**). T cells were subsequently reclustered into 15 clusters (**Supplementary Figure S1F**), and marker-gene expression was used for subtype annotation (**Supplementary Figure S1G**). These clusters were annotated into five functional subtypes: CD4+ naive T cells, CD8+ exhausted T cells (CD8+ Tex), CD8+ tissue-resident memory T cells (CD8+ Trm), effector T cells (Teff), and regulatory T cells (Tregs) (**Figure 2E**). Descriptive comparison according to *H. pylori* status suggested a greater representation of CD8+ Tex cells and Tregs in the *H. pylori*-positive samples (**Figure 2F**). Given the limited number of patients, these compositional patterns should be interpreted descriptively rather than as definitive differences between *H. pylori*-status groups.

**Figure 2:**
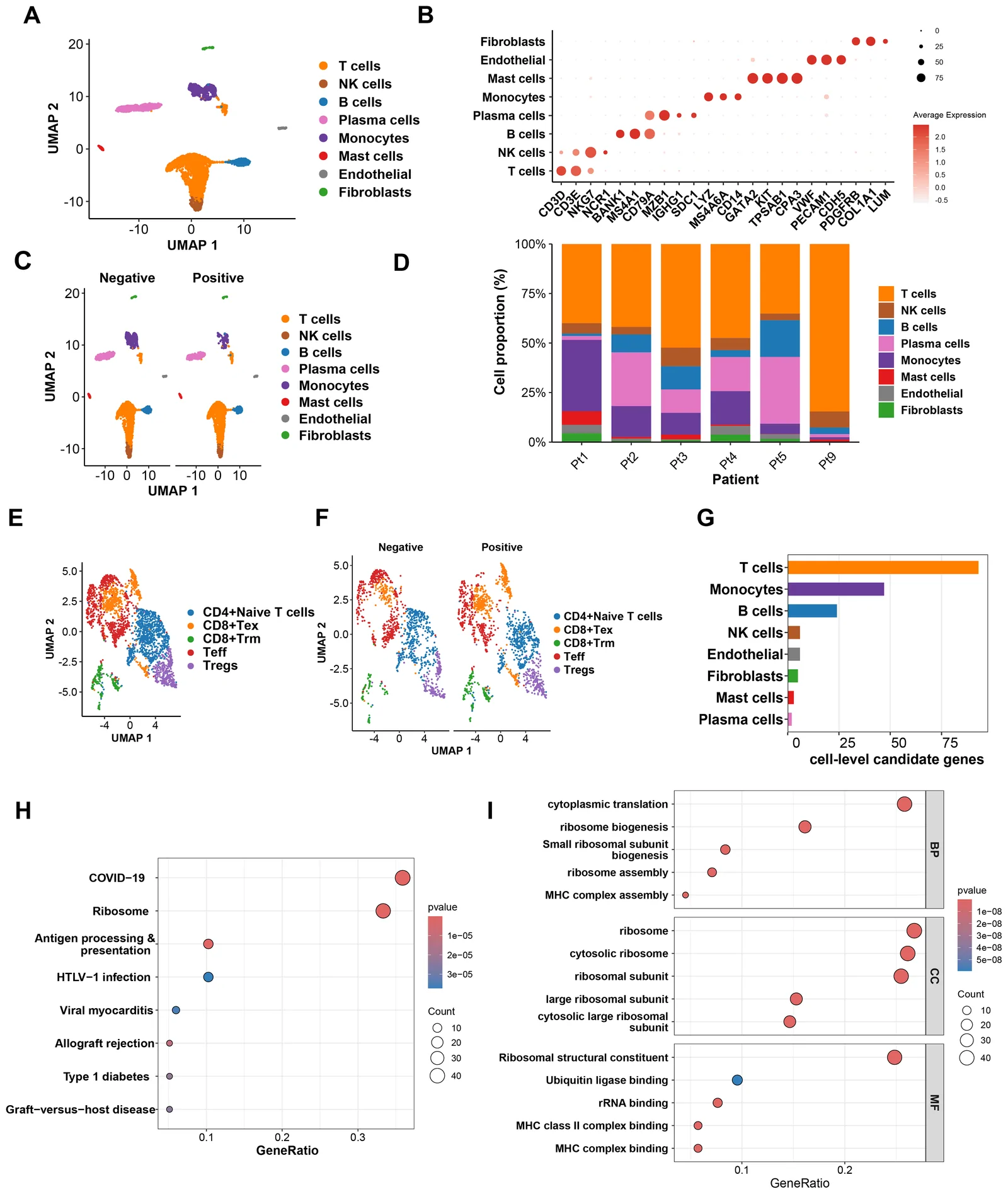
Exploratory differences in the GC immune microenvironment according to *H. pylori* status. **(A)** UMAP projection showing eight major annotated cell types derived from GSE234129. **(B)** Dot plot displaying the expression of canonical marker genes among the annotated cell types. **(C)** UMAP visualization comparing the distribution of major cell types between the *H. pylori-positive* and -negative groups. **(D)** Stacked bar plot showing the relative abundance of each major cell type across the six individual patients. **(E)** UMAP plot showing five annotated T-cell subtypes. **(F)** UMAP visualization comparing T-cell subtype distributions between the *H. pylori-*positive and -negative groups. **(G)** Number of exploratory candidate genes identified according to *H. pylori* status in each major cell type. **(H)** KEGG pathway enrichment analysis of the candidate genes. **(I)** GO enrichment analysis.

### 3.2 Exploratory candidate gene screening according to *H. pylori* status

Exploratory cell-level differential-expression analysis between the three *H. pylori*-positive and three *H. pylori*-negative patients identified 163 exploratory candidate genes showing cell-level expression differences according to *H. pylori* status. These candidate genes were subsequently intersected with genes available in TCGA-STAD for downstream prognostic modelling. The 163 exploratory candidate genes were predominantly distributed in T cells, monocytes, and B cells (**Figure 2G**). Functional enrichment analysis of these candidate genes indicated overrepresentation of immune-related pathways and biological processes. KEGG analysis showed enrichment of pathways including antigen processing and presentation (**Figure 2H**), whereas GO analysis identified terms related to MHC complex assembly, MHC class II complex binding, and other immune-related functions (**Figure 2I**). These findings suggest possible differences in immune-related transcriptional patterns according to *H. pylori* status. To address potential cellular pseudoreplication, we further performed a patient-level T-cell pseudobulk sensitivity analysis. MDS analysis showed no clear separation according to *H. pylori* status (**Supplementary Figure S1H**). Among 9, 046 tested genes, none met the criteria of |log2FC| ≥ 1 and FDR < 0.05, although 78 genes met the exploratory nominal criteria (**Supplementary Table S1**). Accordingly, the cell-level differential-expression and enrichment findings should be considered exploratory and hypothesis-generating.

### 3.3 Development and evaluation of the eight-gene prognostic signature

We constructed a prognostic model in TCGA-STAD using Cox and LASSO regression analyses. From the 163 exploratory candidate genes, univariable Cox analysis retained 38 candidates with *p* < 0.20 for further model development (**Figure 3A**). LASSO Cox regression retained 14 genes with non-zero coefficients at λmin while controlling for model complexity (**Figure 3B**). Subsequent multivariable Cox modelling with stepwise selection finally retained eight genes to form the signature: RGS1, CXCR4, CXCR6, TMSB4X, APOD, RPS4X, RPS5, and RPS18 (**Figure 3C**). Patients stratified by the median risk score showed significantly poorer OS in the high-risk group (*p* < 0.0001, **Figure 3D**). The model yielded 1-, 3-, and 5-year AUCs of 0.72, 0.69, and 0.77, respectively (**Figure 3D**). Risk-score distribution, survival status, and expression patterns of the eight signature genes are shown in **Supplementary Figure S2A**. The signature was further evaluated in GSE26899 and GSE15459, with prognostic stratification observed in both external cohorts, although discrimination varied between datasets (**Figure 3E**; **Supplementary Figure S2B**).

**Figure 3:**
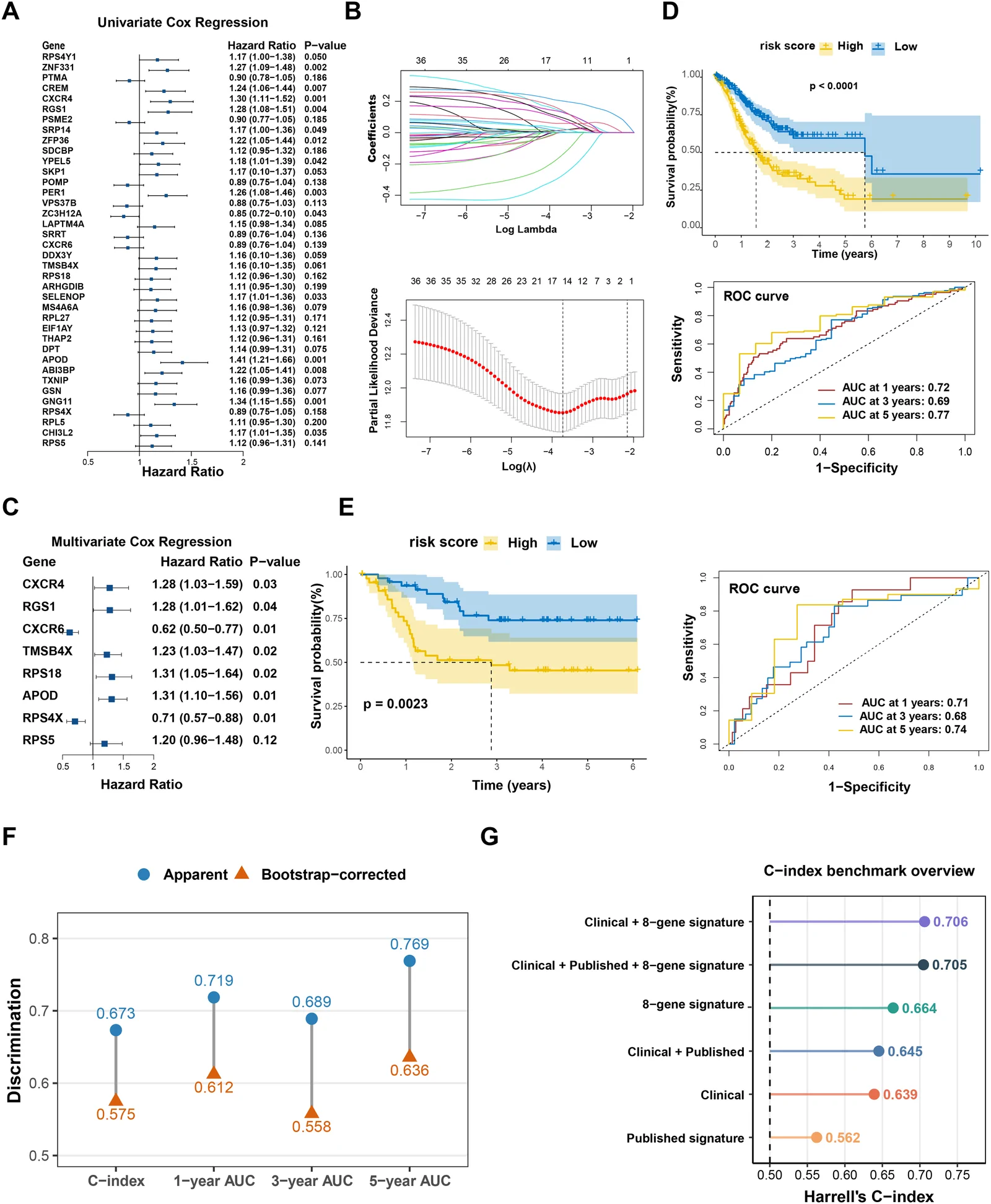
Construction and evaluation of the eight-gene prognostic signature. **(A)** Forest plot of univariable Cox regression analysis of the candidate genes. **(B)** LASSO Cox regression coefficient profiles and selection of the penalty parameter using 10-fold cross-validation. **(C)** Forest plot of the final multivariable Cox regression model. **(D)** Kaplan-Meier survival and time-dependent ROC curves in the TCGA-STAD cohort. **(E)** External evaluation of the prognostic signature using Kaplan-Meier survival and time-dependent ROC analyses in the GSE26899 cohort. **(F)** Comparison of apparent and full-pipeline bootstrap-corrected Harrell’s C-index and 1-, 3-, and 5-year AUCs in TCGA-STAD. **(G)** Comparison of Harrell’s C-index among the clinicopathological model, the published 32-gene signature, the eight-gene signature, and the corresponding combined models.

Full-pipeline bootstrap validation yielded an optimism-corrected C-index of 0.575 and corrected 1-, 3-, and 5-year AUCs of 0.612, 0.558, and 0.636, respectively, indicating limited prognostic discrimination after accounting for model-development optimism. In comparative analyses, the eight-gene signature showed higher discrimination than the published 32-gene signature in the matched TCGA-STAD cohort, while addition of the eight-gene score to clinicopathological variables further improved model discrimination (**Figure 3G**; **Supplementary Figure S2C-F**). We further compared the prognostic performance of RGS1 alone with that of the complete eight-gene signature in the same TCGA-STAD patients. The eight-gene signature showed a higher C-index than RGS1 alone (0.673 vs. 0.574; ΔC-index = 0.099, 95% CI 0.038-0.161, *p* = 0.002; **Supplementary Figure S2G**) and higher 1-, 3-, and 5-year time-dependent AUCs (**Supplementary Figure S2H**). A nested likelihood-ratio test further showed improved model fit after inclusion of the remaining seven genes (χ² = 50.61, df = 7, *p* < 0.001).

Because three components of the signature (RPS4X, RPS5, and RPS18) encode ribosomal proteins, we performed a sensitivity analysis excluding all RPS/RPL genes before repeating the complete modelling procedure. After exclusion of all RPS/RPL genes and repetition of the model-development procedure, all five non-ribosomal components of the original signature (RGS1, CXCR4, CXCR6, TMSB4X, and APOD) were independently reselected. In a complementary fixed-coefficient sensitivity analysis, removal of RPS4X, RPS5, and RPS18 resulted in only modest reductions in apparent discrimination, indicating that the prognostic signal was not solely attributable to the ribosomal components (**Supplementary Table S2**). In the independent *H. pylori*-annotated GSE62254 cohort, the five non-ribosomal components showed directionally higher expression in *H. pylori*-positive tumors and nominal gene-set-level enrichment (CAMERA P = 0.044). However, the composite eight-gene risk score did not differ significantly according to *H. pylori* status (P = 0.574).

### 3.4 Consensus clustering based on the eight-gene signature

Consensus clustering of TCGA-STAD samples based on the expression of the eight signature genes identified three expression-based clusters, with clustering stability assessed using consensus matrices and cumulative distribution function curves (**Supplementary Figures S3A-C**). PCA showed separation among Cluster 1, Cluster 2, and Cluster 3 (**Figure 4A**). Survival analysis demonstrated significant differences in OS among the three clusters (*p* = 0.0089, **Figure 4B**), with Cluster 1 showing relatively favorable survival compared with Clusters 2 and 3. These findings indicate that expression-based clustering using the eight-gene signature was associated with distinct survival patterns in TCGA-STAD.

**Figure 4:**
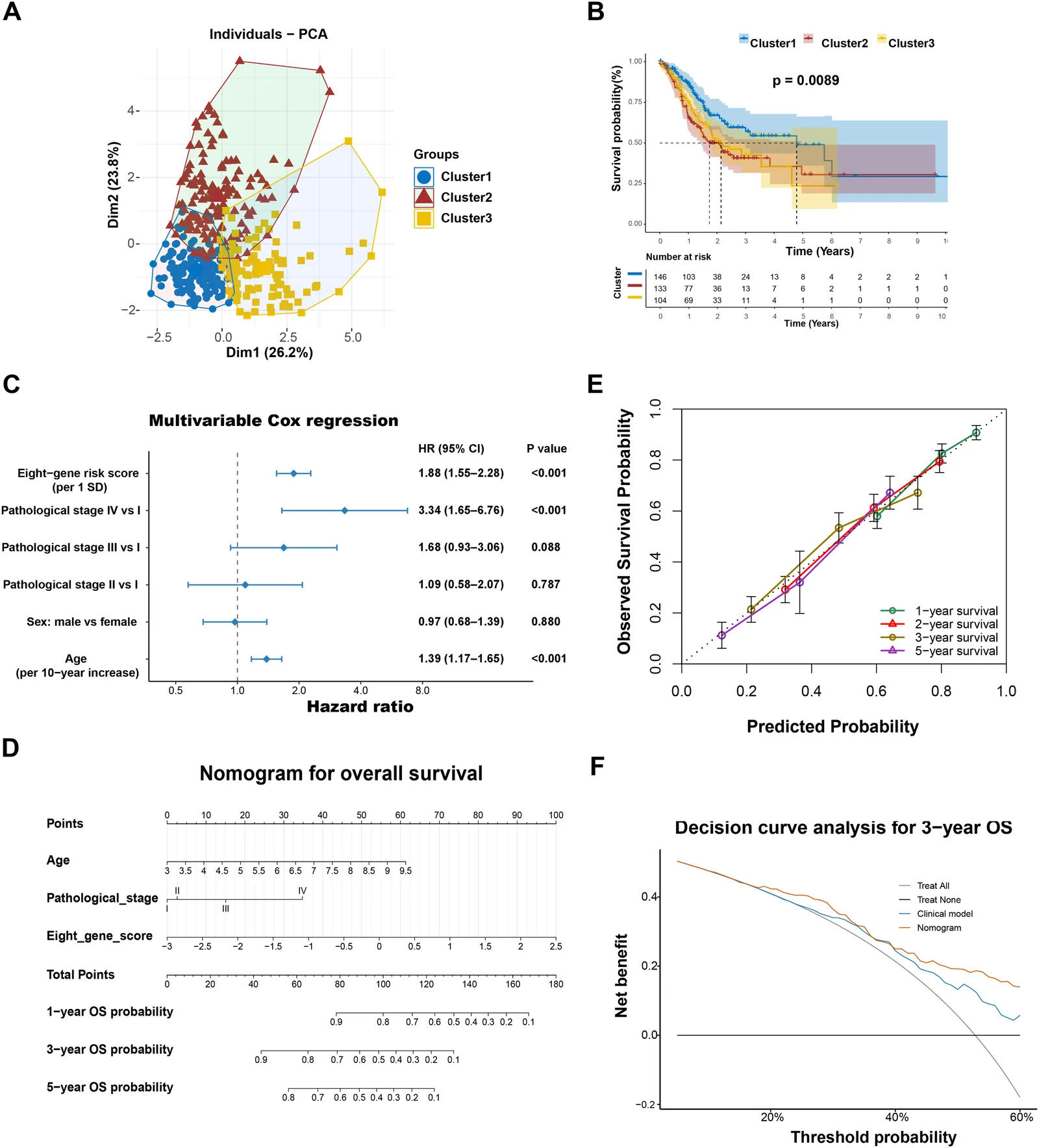
Molecular subtyping and construction of the prognostic nomogram. **(A)** PCA plot illustrating the distribution of Clusters 1, 2, and 3. **(B)** Kaplan-Meier survival curves showing significant differences in OS among the three clusters (p = 0.0089). **(C)** Multivariable Cox regression analysis of OS in the TCGA-STAD cohort. **(D)** Prognostic nomogram integrating age, pathological stage, and the eight-gene risk score to estimate 1-, 3-, and 5-year OS probabilities. **(E)** Calibration curves comparing predicted and observed OS probabilities. **(F)** Decision curve analysis of the nomogram for 3-year OS in TCGA-STAD.

### 3.5 Nomogram construction and evaluation

Univariable Cox regression showed that the eight-gene risk score, age, and pathological stages III and IV were significantly associated with OS, whereas sex and pathological stage II were not statistically significant (**Supplementary Figure S3D**). Multivariable Cox regression further showed that the eight-gene risk score remained independently associated with OS after adjustment for age, sex, and pathological stage (**Figure 4C**). A nomogram integrating the eight-gene risk score, age, and pathological stage was subsequently constructed to estimate 1-, 3-, and 5-year OS probabilities (**Figure 4D**). Calibration analysis showed agreement between predicted and observed survival probabilities (**Figure 4E**). For 3-year OS, decision curve analysis showed that the nomogram provided greater net benefit than the clinicopathological model across a range of threshold probabilities (**Figure 4F**). The nomogram had a C-index of 0.70 (95% CI 0.65-0.74), indicating moderate discriminative ability.

### 3.6 Genomic and pathway characteristics associated with the eight-gene signature

To characterize genomic and pathway features associated with the eight-gene signature, we compared molecular characteristics between the high- and low-risk groups in TCGA-STAD. The low-risk group showed significantly higher TMB than the high-risk group (*p* < 0.001), with a greater proportion of TMB-high cases in the low-risk group (**Figure 5A**). Somatic mutation profiling further showed a higher overall proportion of mutated samples in the low-risk group than in the high-risk group (95.79% vs. 88.95%), with TTN and TP53 among the most frequently mutated genes in both groups (**Supplementary Figures S3E, F**). Risk scores were also significantly higher in microsatellite-stable (MSS) tumors than in microsatellite-instability (MSI) tumors (*p* < 0.001; **Figure 5B**). KEGG enrichment analysis of differentially expressed genes between the two risk groups identified pathways related to neuroactive ligand-receptor interaction, cytoskeletal organization, calcium signaling, cell adhesion molecules, cAMP/cGMP signaling, and ECM-receptor interaction (**Figure 5C**). GSVA further showed distinct pathway-activity patterns between the two groups. Higher risk scores were associated with increased activity of MYOGENESIS, EPITHELIAL-MESENCHYMAL TRANSITION, ANGIOGENESIS, HEDGEHOG SIGNALING, UV RESPONSE DN, APICAL JUNCTION, and COAGULATION, whereas the low-risk group showed relatively higher activity of pathways including G2M CHECKPOINT, E2F TARGETS, MYC TARGETS, MTORC1 SIGNALING, PEROXISOME, and DNA REPAIR (**Figure 5D**). Consistent with these findings, GSEA showed enrichment of several signaling and extracellular-interaction pathways, including calcium signaling, cell adhesion molecules, complement and coagulation cascades, ECM-receptor interaction, focal adhesion, gap junction, Hedgehog signaling, and PPAR signaling (**Figure 5E**). Together, these analyses indicate that the eight-gene risk score is associated with differences in genomic features and pathway-level transcriptional programs in gastric cancer.

**Figure 5:**
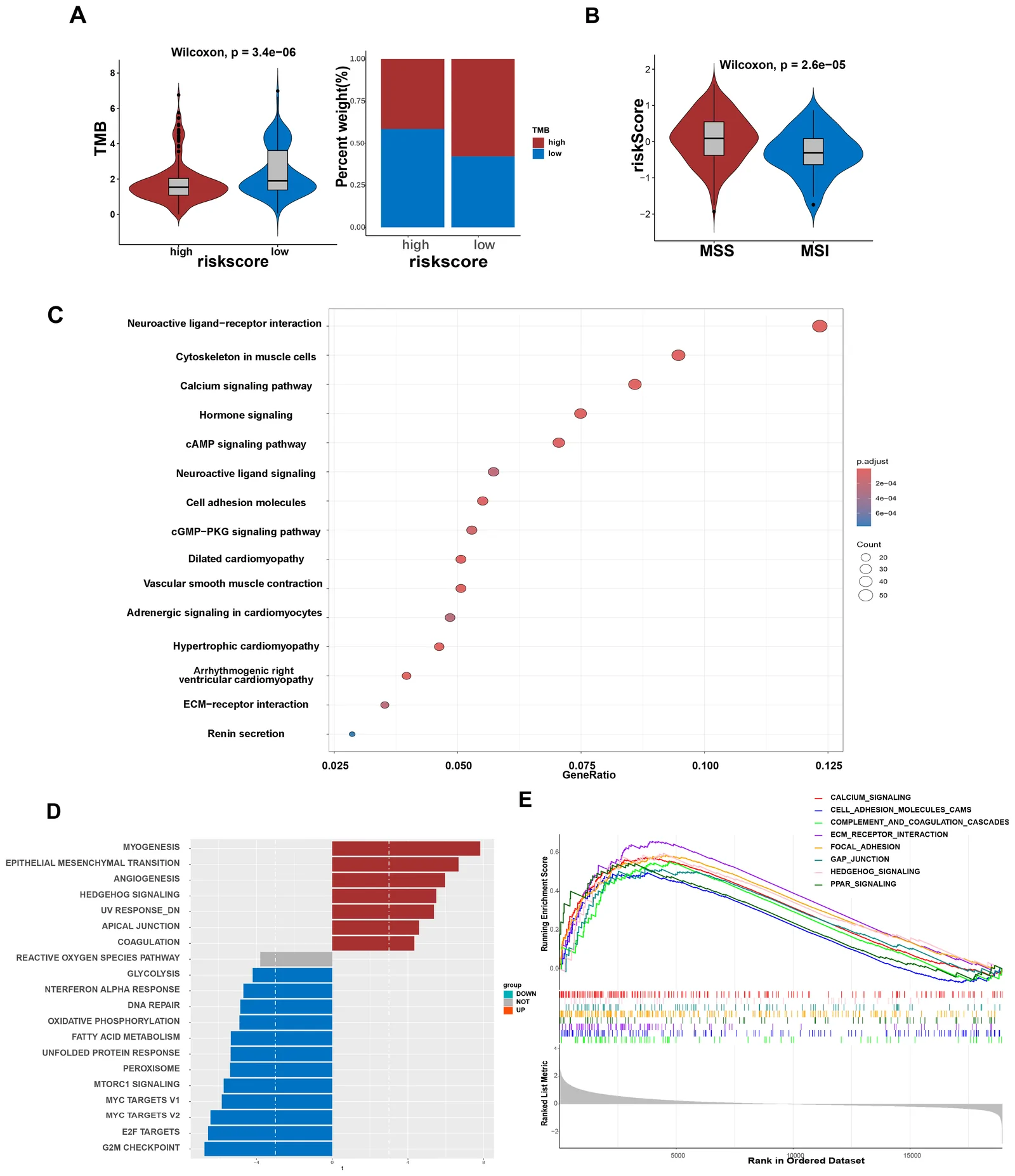
Genomic and pathway characteristics associated with the eight-gene signature. **(A)** Comparison of tumor mutation burden (TMB) between the high- and low-risk groups and the proportions of TMB-high and TMB-low cases in each group. **(B)** Comparison of risk scores between microsatellite-stable (MSS) and microsatellite-instability (MSI) tumors. **(C)** KEGG pathway enrichment analysis of differentially expressed genes between the high- and low-risk groups. **(D)** GSVA showing differences in Hallmark pathway activities between the two risk groups. **(E)** GSEA showing representative pathways enriched according to the eight-gene risk score.

### 3.7 Immune associations and exploratory immunotherapy-related analyses

To assess how the eight-gene risk score was associated with the tumor immune microenvironment, we evaluated immune-cell infiltration, stromal and immune features, and exploratory immunotherapy-related indicators. Low-risk tumors showed higher levels of several immune-cell populations, including plasma cells, CD8+ T cells, activated CD4+ memory T cells, and follicular helper T cells (**Figure 6A**). The risk score was positively correlated with the stromal score (r = 0.37, *p* < 0.001; **Figure 6B**), whereas no significant correlation was observed with the immune score (r = 0.03, p = 0.572; **Figure 6C**). Immunophenoscore (IPS) analysis showed no significant difference between the low- and high-risk groups in the PD-1−/CTLA-4− or PD-1−/CTLA-4+ categories, whereas higher IPS values were observed in the low-risk group in the PD-1+/CTLA-4− (*p* = 0.002) and PD-1+/CTLA-4+ (p < 0.001) categories (**Figure 6D**). TIDE analysis showed significantly higher overall TIDE and exclusion scores in the high-risk group, while the difference in dysfunction score did not reach statistical significance (*p* = 0.051; **Figure 6E**). These computational findings indicate that the eight-gene risk score was associated with several immunotherapy-related immune features, but do not directly establish differential treatment responsiveness. We therefore further examined observed anti-PD-1 response in the disease-matched PRJEB25780 gastric cancer cohort. The eight-gene risk score did not differ significantly between responders and non-responders (*p* = 0.617; **Figure 6F**), and response rates were also not significantly different between the high- and low-risk groups (*p* = 0.314; **Figure 6G**). In the IMvigor210 urothelial carcinoma cohort, patients with a high eight-gene risk score showed shorter OS than those with a low risk score (*p* = 0.039; **Supplementary Figure S3G**); given the cross-tumor nature of this cohort, this finding was interpreted as exploratory rather than as gastric cancer-specific validation.

**Figure 6:**
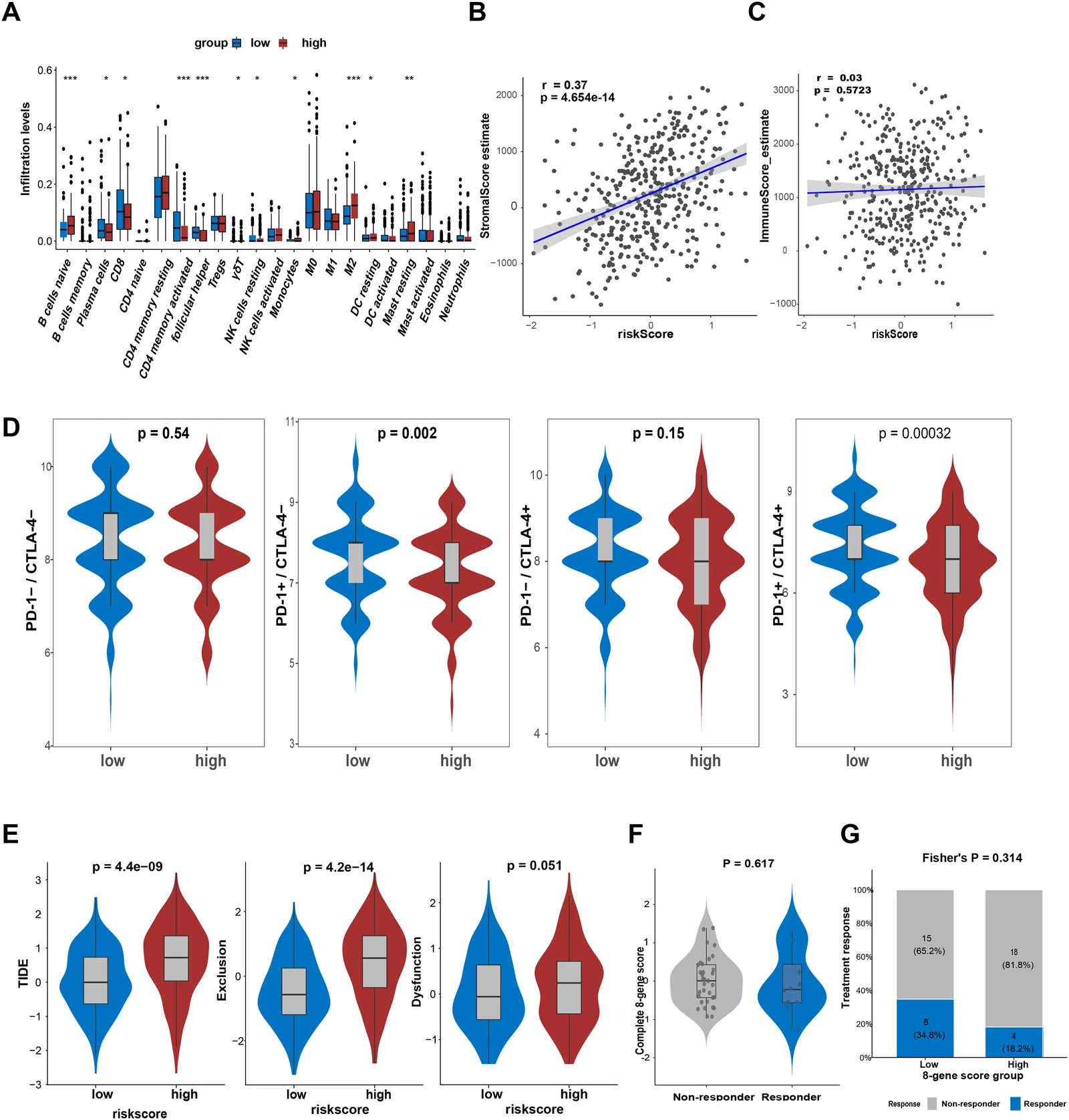
Immune-cell infiltration and exploratory immunotherapy-related analyses. **(A)** Immune-cell infiltration profiles in the high- and low-risk groups estimated using CIBERSORT. **(B)** Correlation between the eight-gene risk score and stromal score. **(C)** Correlation between the eight-gene risk score and immune score. **(D)** Immunophenoscore comparisons between the high- and low-risk groups according to PD-1 and CTLA-4 status. **(E)** Comparison of TIDE, exclusion, and dysfunction scores between the two risk groups. **(F)** Comparison of the complete eight-gene risk score between anti-PD-1 responders and non-responders in the PRJEB25780 gastric cancer cohort. **(G)** Proportions of responders and non-responders in the high- and low-risk groups in PRJEB25780. *p < 0.05, **p < 0.01, and ***p < 0.001.

### 3.8 Expression patterns and immune associations of the eight-gene signature in gastric cancer

Through analysis of TCGA-STAD data, RGS1 expression was higher in gastric tumor tissues than in normal tissues and differed across pathological stages (*p* = 0.0071; **Figure 7A**). In contrast, APOD expression was lower in tumor tissues than in normal tissues and also differed across pathological stages (*p* = 0.0034; **Figure 7B**). Single-cell transcriptomic analysis further showed that RGS1 was predominantly expressed in immune cells, particularly T cells, whereas APOD expression was mainly observed in fibroblasts (**Figure 7C**). The remaining six signature genes, including CXCR4, CXCR6, RPS4X, TMSB4X, RPS5, and RPS18, showed distinct cell-type expression patterns (**Supplementary Figures S4A-F**). Given the predominant expression of RGS1 in T cells, we further characterized transcriptional and predicted intercellular-interaction features associated with RGS1-expressing T cells. CellChat analysis indicated that RGS1+ T cells participated in multiple predicted ligand-receptor interaction networks with B cells, fibroblasts, endothelial cells, and other cell populations (**Figure 7D**). Pathway analysis showed enrichment of several immune- and signaling-related processes, including chemokine receptor-chemokine binding, GPCR signaling, interleukin signaling, and cytokine signaling (**Figure 7E**). Predicted ligand-receptor analysis further identified several interactions involving RGS1+ T cells, including the MIF-(CD74+CXCR4) interaction (**Figure 7F**). These findings represent transcriptionally inferred interaction patterns rather than direct evidence of functional cell-cell communication. We next explored the associations of individual signature genes with observed anti-PD-1 response in the PRJEB25780 gastric cancer cohort. RGS1 expression was higher in responders than in non-responders (*p* = 0.0247; **Figure 7G**). RGS1 expression was also positively correlated with the complete eight-gene risk score (Spearman r = 0.414, *p* = 0.0048; **Figure 7G**). In contrast, APOD expression was lower in responders than in non-responders (*p* = 0.0123; **Supplementary Figure S4G**). Given the limited sample size of the immunotherapy cohort, these gene-level associations should be interpreted cautiously and require validation in larger disease-matched cohorts.

**Figure 7:**
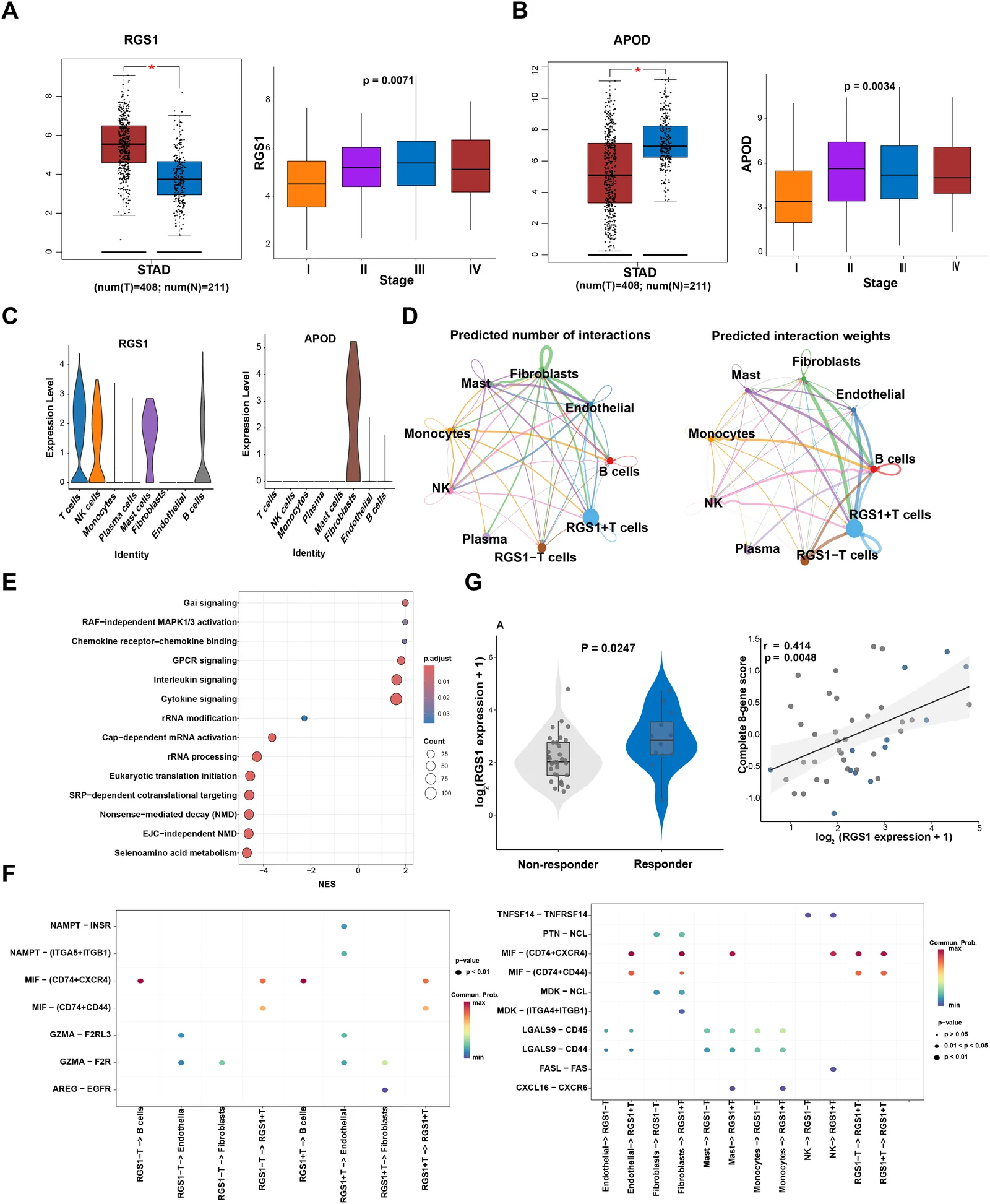
Expression patterns and immune associations of the eight-gene signature in gastric cancer. **(A, B)** Differential expression of RGS1 and APOD between gastric tumor and normal tissues and their associations with pathological stage in TCGA-STAD. **(C)** Expression profiles of RGS1 and APOD across major immune and stromal cell types in the GSE234129 single-cell dataset. **(D)** CellChat-predicted numbers and weights of intercellular interactions involving RGS1+ T cells and other cell types. **(E)** Pathway enrichment analysis of RGS1+ T cells. **(F)** Predicted ligand-receptor interactions involving RGS1+ T cells as signal senders or receivers, including MIF-(CD74+CXCR4). **(G)** RGS1 expression in anti-PD-1 responders and non-responders and its correlation with the complete eight-gene risk score in the PRJEB25780 cohort.

### 3.9 Tissue-level validation of RGS1 expression and its clinical and immune associations

To evaluate the tissue-level expression and clinical relevance of RGS1, IHC analysis was performed in 128 patients with GC and known *H. pylori* status. Postoperative disease progression was more frequent in patients with *H. pylori* positivity (*p* = 0.002, **Table 1**). RGS1 protein expression was higher in gastric cancer tissues than in adjacent normal mucosa. It was also higher in *H. pylori*-positive tumors than in *H. pylori*-negative tumors (**Figure 8A**). Kaplan-Meier analysis showed that patients with high RGS1 expression had shorter DFS and OS than those with low RGS1 expression. The differences were significant for both DFS (*p* = 0.0012, **Figure 8B**) and OS (*p* = 0.00014, **Figure 8C**). We next examined the combined association of *H. pylori* status and RGS1 expression with survival. Patients were divided into four groups: HP−/RGS1-Low, HP−/RGS1-High, HP+/RGS1-Low, and HP+/RGS1-High. Significant survival differences were observed among the four groups. This was found for both DFS and OS (both *p* < 0.0001; **Supplementary Figure S4H** and **Figure 8D**). The HP+/RGS1-High group showed the least favorable survival pattern. Univariable Cox analysis showed that high RGS1 expression, pathological stage III, poor differentiation, and *H. pylori* positivity were associated with higher mortality risk (**Supplementary Figure S4I**). Perioperative treatment was associated with lower mortality risk. In the multivariable model, high RGS1 expression remained independently associated with OS (HR = 2.36, 95% CI 1.36-4.10, *p* = 0.002; **Figure 8E**). In the complete-case multivariable analysis (n = 126), *H. pylori* positivity was independently associated with poorer OS (HR = 2.28, 95% CI 1.20-4.30, P = 0.011). After exclusion of the 14 patients whose *H. pylori* status was determined by serology alone, the association was slightly attenuated but remained statistically significant (HR = 2.15, 95% CI 1.11–4.17, P = 0.024; **Supplementary Table S3**). The association between high RGS1 expression and poorer OS also remained stable in this sensitivity analysis (HR = 2.29, 95% CI 1.28-4.10, P = 0.005). Age, poor differentiation, and perioperative treatment were not statistically significant in the multivariable model. IHC-based immune profiling showed differences in selected immune-cell markers between the RGS1-high and RGS1-low groups. RGS1-high tumors had higher CD163+ macrophage staining and higher FOXP3+ regulatory T-cell staining, together with lower CD8+ T-cell staining (**Figure 8F**). These findings support an association of high RGS1 expression with poorer survival and distinct immune-cell features at the tissue level.

**Table 1:** Baseline characteristics of patients with and without postoperative disease progression.

Characteristic	Non-progression(n = 48)	Progression(n=80)	*P*
*H. pylori*			0.002
Negative	30 (62.5%)	27 (33.8%)	
Positive	18 (37.5%)	53 (66.3%)	
Age			0.392
≤65 years	16 (33.3%)	21 (26.3%)	
>65 years	32 (66.7%)	59 (73.8%)	
Gender			0.825
Male	37 (77.1%)	63 (78.8%)	
Female	11 (22.9%)	17 (21.3%)	
HER2			0.347
Negative	42 (87.5%)	74 (92.5%)	
Positive	6 (12.5%)	6 (7.5%)	
Grade			0.038
G1	13 (27.1%)	8 (10.0%)	
G2	12 (25.0%)	22 (27.5%)	
G3	23 (47.9%)	50 (62.5%)	
Stage			<0.001
Stage I	14 (29.2%)	6 (7.5%)	
Stage II	16 (33.3%)	20 (25.0%)	
Stage III	18 (37.5%)	54 (67.5%)	
Location			0.825
Proximal	10 (20.8%)	18 (22.5%)	
Distal	38 (79.2%)	62 (77.5%)	
Lauren			0.461
Intestinal	23 (47.9%)	31 (38.8%)	
Diffuse	18 (37.5%)	39 (48.8%)	
Mixed	7 (14.6%)	10 (12.5%)	

**Figure 8:**
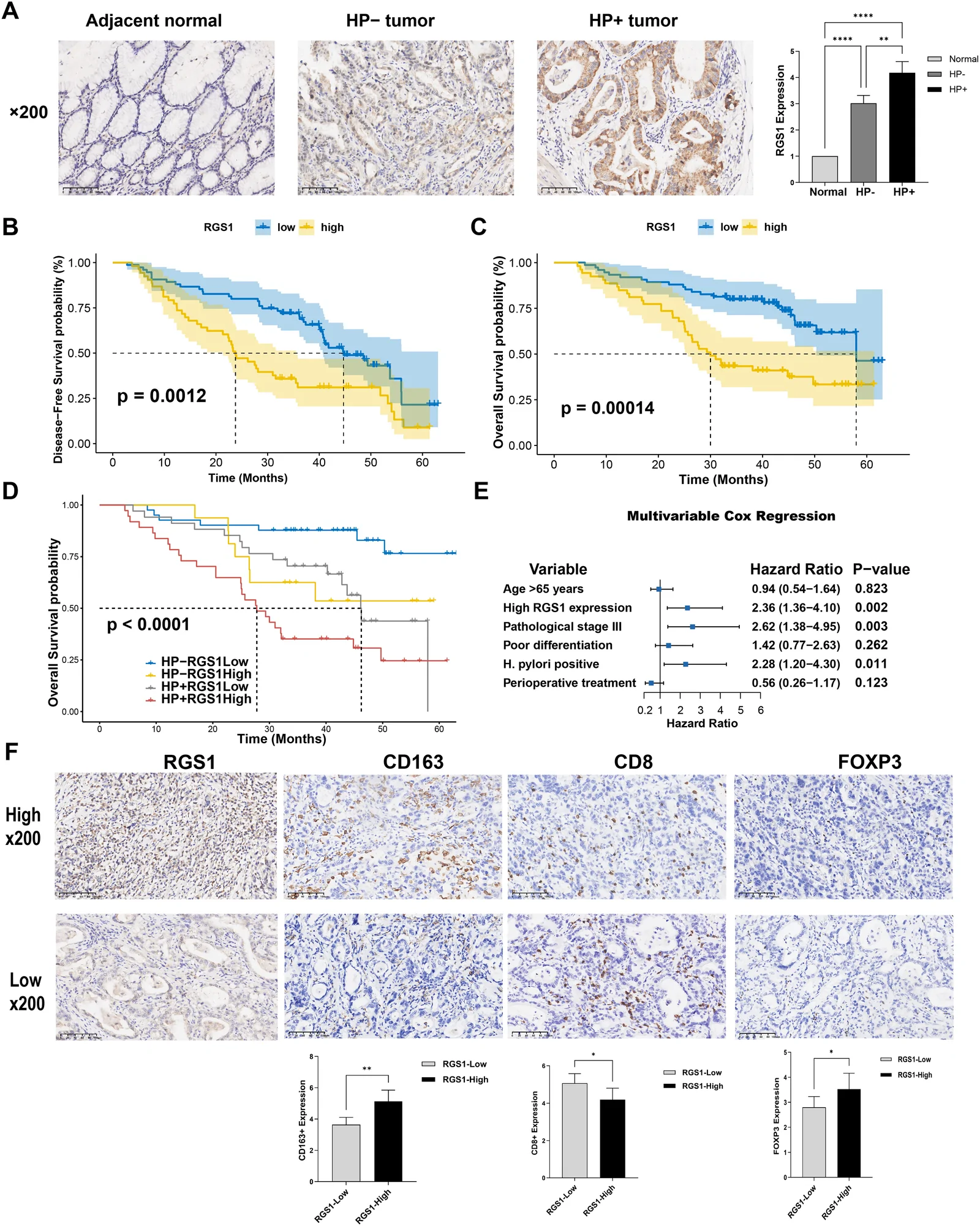
RGS1 expression according to *H. pylori* status and its associations with survival and immune-cell markers. **(A)** Representative immunohistochemical staining and quantitative comparison of RGS1 expression in adjacent normal tissues, *H. pylori-negative* tumors, and *H. pylori-positive* tumors. **(B, C)** Kaplan-Meier curves comparing disease-free survival **(B)** and overall survival **(C)** between the RGS1-high and RGS1-low groups. **(D)** Overall survival according to the combined *H. pylori* status and RGS1-expression groups. **(E)** Multivariable Cox regression analysis of factors associated with overall survival. **(F)** Representative immunohistochemical staining and quantitative comparisons of CD163+, CD8+, and FOXP3+ cells between the RGS1-high and RGS1-low groups. Original magnification, ×200. *p < 0.05, **p < 0.01, and ****p < 0.0001.

## 4 Discussion

GC is a heterogeneous malignancy influenced by both molecular alterations and the tumor immune microenvironment (35). Although *H. pylori* is an established etiological factor in gastric carcinogenesis, its immunological relevance after malignant transformation remains less clear (36, 37).

The single-cell analysis suggested differences in immune-cell composition according to *H. pylori* status. *H. pylori*-positive samples showed relatively higher proportions of T cells, including CD8+ exhausted T cells and Tregs. The exploratory candidate genes were also enriched in antigen-presentation and MHC-related processes. These observations are broadly consistent with previous studies showing that persistent *H. pylori* infection is associated with complex immune regulation and altered immune-cell states (38, 39). However, only six patients were included, and no genes remained significant after FDR correction in the patient-level T-cell pseudobulk analysis. In the independent GSE62254 cohort, the five non-ribosomal components showed directionally higher expression in *H. pylori*-positive tumors and nominal gene-set-level enrichment (CAMERA P = 0.044). These findings support only a limited exploratory association with *H. pylori* status and should not be interpreted as evidence that the final prognostic model is *H. pylori*-specific.

The eight-gene signature showed prognostic stratification in TCGA-STAD and two independent GEO cohorts. Comparative analyses further evaluated its performance relative to clinicopathological variables, a published gastric cancer signature, and RGS1 alone. The complete signature showed better discrimination than RGS1 alone, suggesting that RGS1 does not capture all prognostic information represented by the multigene model. However, bootstrap correction resulted in more modest discrimination than the apparent estimates. Accordingly, the signature should be regarded as an exploratory survival-associated molecular score rather than a clinically validated prognostic tool. Its potential incremental value requires confirmation in prospectively designed cohorts. Three components of the original signature, RPS4X, RPS5, and RPS18, are ribosomal protein genes and may reflect general transcriptional, translational, or cellular-composition effects that are not necessarily disease-specific. Sensitivity analyses showed that all five non-ribosomal components were reselected after removal of RPS/RPL genes from the candidate pool, while fixed deletion of the three ribosomal components produced only modest changes in apparent discrimination. These findings suggest that the model was not entirely dependent on ribosomal-gene signals, although the biological specificity of these components remains uncertain. Several genes within the signature have also been previously associated with tumor progression and immune regulation. CXCR4 and CXCR6 participate in chemokine-mediated immune-cell trafficking, whereas TMSB4X is involved in cytoskeletal remodeling and cell migration (40–42). However, the individual contribution of each component gene requires further investigation, as the prognostic value of this model likely reflects the combined effect of multiple biological features. This interpretation is also consistent with the broader concern that molecular prognostic models should demonstrate incremental value beyond established clinicopathological factors before clinical implementation.

Genomic and immune analyses showed additional differences between the risk groups. Low-risk tumors had higher TMB and were more frequently associated with MSI-related features. Higher risk scores were associated with stromal features and with pathways including EMT, angiogenesis, Hedgehog signaling, and coagulation. TMB and MSI have been widely studied as immunotherapy-related biomarkers (43, 44), while EMT and stromal activation have been associated with immune exclusion and treatment resistance (45–48). TIDE and IPS also showed differences between the risk groups. However, the eight-gene risk score was not significantly associated with observed anti-PD-1 response in PRJEB25780. Therefore, these computational features should not be interpreted as evidence that the signature predicts ICI response. This caution is particularly relevant because previous studies have also reported inconsistent associations between *H. pylori* status and immunotherapy outcomes in GC (49, 50).

RGS1 has been implicated in the regulation of chemokine receptor signaling and T-cell trafficking, and elevated RGS1 expression has been associated with dysfunctional T-cell states in chronic infections and cancer (51). Our single-cell results similarly showed predominant RGS1 expression in T cells and predicted interactions involving RGS1+ T cells, including MIF-(CD74+CXCR4). MIF/CD74 signaling has also been implicated in tumor immune regulation (52–54). At the tissue level, high RGS1 expression was independently associated with poorer OS and was accompanied by higher CD163+ and FOXP3+ staining and lower CD8+ staining. These findings are also consistent with recent evidence linking RGS1 expression with adverse prognosis and macrophage-associated immune features in GC (55). However, our study differs in additionally examining *H. pylori* status, T-cell-associated expression patterns, and the prognostic contribution of RGS1 relative to the complete eight-gene signature. The present data support association rather than a direct mechanistic role for RGS1. Interestingly, RGS1 expression was higher, whereas APOD expression was lower, in anti-PD-1 responders in PRJEB25780. This differs from the adverse prognostic association of high RGS1 expression. Prognostic and treatment-response biomarkers are not necessarily equivalent, and RGS1 expression and the multigene risk score may capture different biological features. Given the small immunotherapy cohort, these findings remain exploratory.

Several other limitations should also be considered. The model was derived from retrospective datasets, and its optimism-corrected discrimination was limited, restricting conclusions regarding clinical predictive utility. The single-cell dataset was small, and the IHC cohort was from a single center. Different clinical methods were used to determine *H. pylori* status. Although the association between *H. pylori* positivity and OS was slightly attenuated after exclusion of serology-only cases, it remained statistically significant; nevertheless, the single-center design and heterogeneous diagnostic methods remain important limitations. IMvigor210 was a cross-tumor exploratory cohort and cannot provide disease-specific validation. Finally, CellChat and pathway analyses are computational and do not establish mechanism. Prospective multicenter validation and functional studies of RGS1, including T-cell migration and ligand-receptor interaction experiments, are therefore warranted. EBV status and PD-L1 combined positive score were unavailable in our analyzed cohorts, and therefore could not be incorporated into direct comparative analyses.

## 5 Conclusion

In conclusion, we developed an eight-gene survival-associated prognostic signature and characterized its clinical, genomic, and immune associations in gastric cancer. *H. pylori*-related analyses provided exploratory biological context but did not establish *H. pylori* specificity of the final model. The signature showed limited optimism-corrected discrimination and should therefore be considered exploratory rather than clinically validated. RGS1 showed additional associations with poorer survival and distinct immune-cell features, warranting further prospective and functional investigation.

## Data Availability

The publicly available datasets analyzed in this study are available from the following repositories: TCGA-STAD from the Genomic Data Commons (https://www.cancer.gov/tcga); GSE234129, GSE26899, GSE15459, and GSE62254 from the Gene Expression Omnibus (https://www.ncbi.nlm.nih.gov/geo/); and PRJEB25780 from the European Nucleotide Archive (https://www.ebi.ac.uk/ena/browser/view/PRJEB25780). The processed IMvigor210 data were obtained from the IMvigor210CoreBiologies R package, with raw sequencing data available through the European Genome-phenome Archive under accession EGAS00001002556. The de-identified clinicopathological and immunohistochemical data generated in the present study are not publicly available because the participant consent and ethics approval did not permit unrestricted public disclosure. These data may be obtained from the corresponding author (47100214@hebmu.edu.cn) upon reasonable request and subject to institutional ethics approval and applicable privacy regulations. All other data supporting the findings of this study are included in the article and its **Supplementary Material**.
