# Supplementary material for: An eight-gene prognostic signature and its immune associations in gastric cancer: integrated transcriptomic and clinical analyses
Source: Front Oncol. 2026 Oct 22;16:1930319. doi: 10.3389/fonc.2026.1930319 (PMC13735604; doi:10.3389/fonc.2026.1930319)
Supplement: Supplementary file 1 [file DataSheet1.pdf]

Supplementary Material

Supplementary Figures

Figure S1. Quality control, clustering, and patient-level sensitivity analysis of the single-cell dataset. (A) Quality-control metrics for the major cell types, including RNA counts, detected features, mitochondrial transcript percentage, and hemoglobin transcript percentage. (B) PCA plot showing interpatient variation. (C) Identification of the top 2,000 highly variable genes. (D) t-SNE plot showing the initial 17 cell clusters. (E) Relative proportions of the eight major cell types according to *H. pylori* status. (F) UMAP plot showing 15 T-cell clusters. (G) Dot plot of marker-gene expression used for T-cell subtype annotation. (H) MDS plot of patient-level T-cell pseudobulk expression profiles according to *H. pylori* status.

Figure S2. Additional evaluation and comparative analysis of the eight-gene prognostic signature. (A) Distribution of risk scores, survival status, and expression patterns of the eight signature genes in TCGA-STAD. (B) Kaplan-Meier survival and time-dependent ROC analyses of the eight-gene signature in the GSE15459 cohort. (C) Comparison of discrimination among the clinicopathological model, the eight-gene model, and their combined model. (D) Apparent and bootstrap-corrected discrimination estimates for the clinicopathological and combined models. (E) Bootstrap distribution of the difference in Harrell's C-index between the combined and clinicopathological models. (F) Comparison of 1-, 3-, and 5-year time-dependent AUCs among the published 32-gene signature, the eight-gene signature, and the corresponding combined models. (G) Comparison of Harrell's C-index between RGS1 alone and the complete eight-gene signature. (H) Comparison of 1-, 3-, and 5-year time-dependent AUCs between RGS1 alone and the complete eight-gene signature.

Figure S3. Consensus clustering, mutation profiles, and exploratory cross-tumor evaluation. (A) t-SNE visualization of the three expression-based clusters. (B) Consensus matrix for the three-cluster solution. (C) Consensus cumulative distribution function curves. (D) Univariable Cox regression analysis of the eight-gene risk score and clinicopathological variables. (E, F) Somatic mutation profiles of the low-risk (E) and high-risk (F) groups. (G) Kaplan-Meier survival analysis according to the eight-gene risk score in the IMvigor210 urothelial carcinoma cohort.

Figure S4. Cell-type expression patterns and additional clinical analyses of the signature genes. (A-F) Expression patterns of CXCR4, CXCR6, RPS4X, TMSB4X, RPS5, and RPS18 across the major cell types in the GSE234129 single-cell dataset. (G) APOD expression in anti-PD-1 responders and non-responders in the PRJEB25780 cohort. (H) Disease-free survival according to the combined *H. pylori* status and RGS1-expression groups. (I) Univariable Cox regression analysis of factors associated with overall survival in the immunohistochemical cohort.

Figure S1

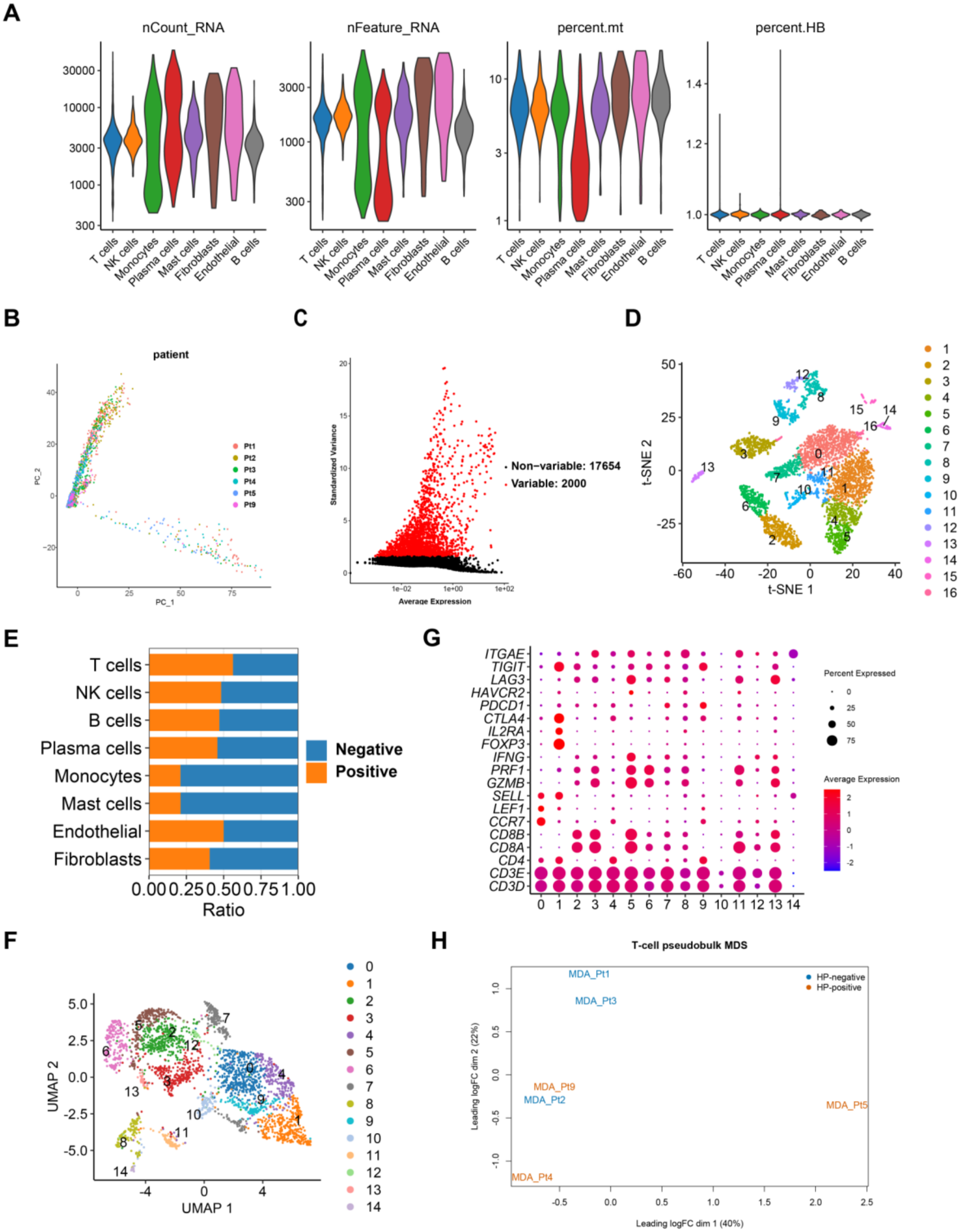
A
nCount_RNA
nFeature_RNA
percent.mt
percent.HB
T cells
NK cells
Monocytes
Plasma cells
Mast cells
Fibroblasts
Endothelial
B cells
B
patient
PC_1
PC_2
Pt1
Pt2
Pt3
Pt4
Pt5
Pt9
C
Standardized Variance
Average Expression
Non−variable: 17654
Variable: 2000
D
t-SNE 1
t-SNE 2
E
Negative
Positive
Ratio
G
ITGAE
TIGIT
LAG3
HAVCR2
PDCD1
CTLA4
IL2RA
FOXP3
IFNG
PRF1
GZMB
SELL
LEF1
CCR7
CD8B
CD8A
CD4
CD3E
CD3D
Percent Expressed
Average Expression
F
UMAP 1
UMAP 2
H
T-cell pseudobulk MDS
HP-negative
HP-positive
MDA_Pt1
MDA_Pt3
MDA_Pt9
MDA_Pt2
MDA_Pt5
MDA_Pt4
Leading logFC dim 1 (40%)
Leading logFC dim 2 (22%)

Figure S2

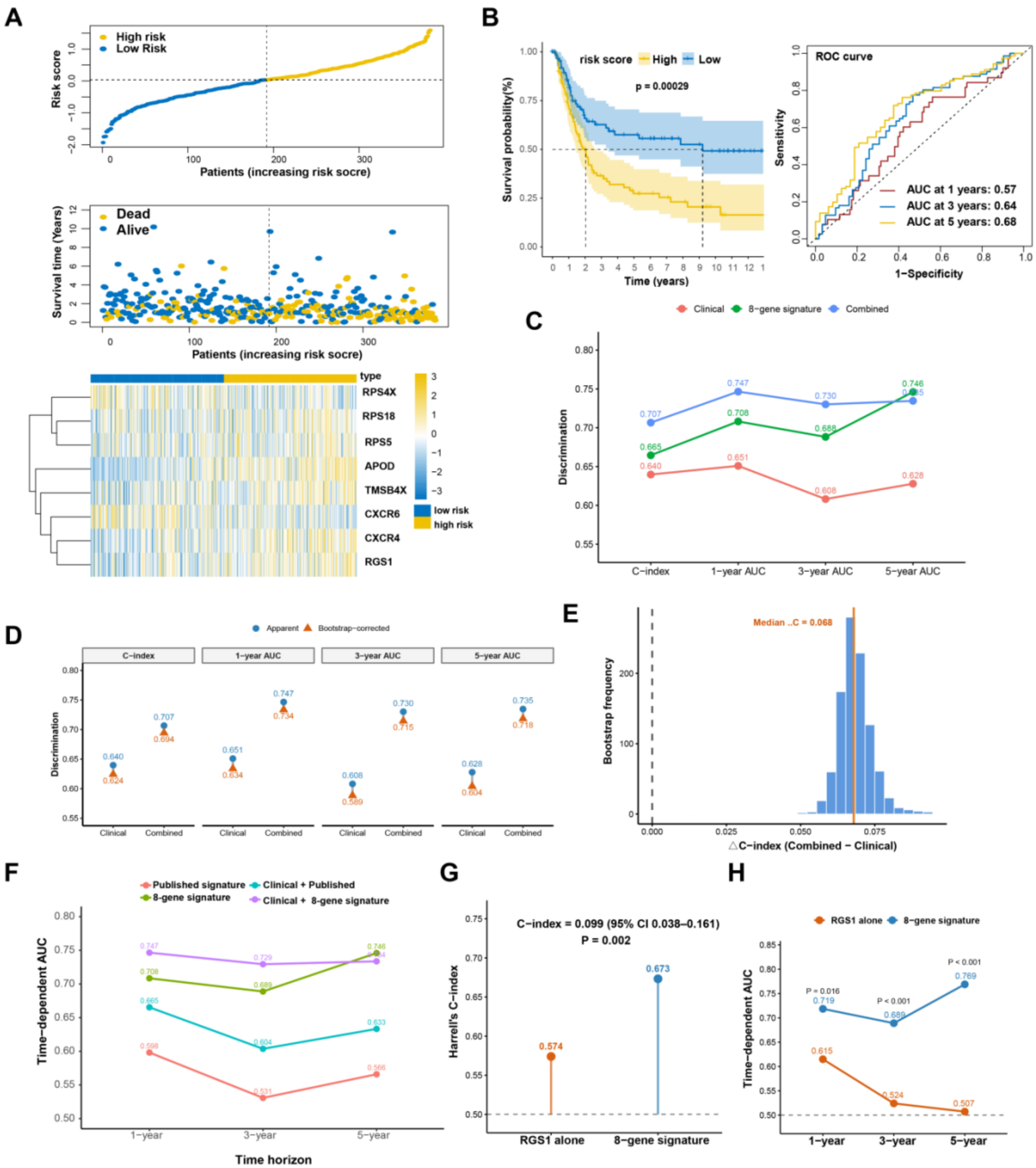

A
High risk
Low Risk
Risk score
Patients (increasing risk socre)
Dead
Alive
Survival time (Years)
Patients (increasing risk socre)
type
RPS4X
RPS18
RPS5
APOD
TMSB4X
CXCR6
CXCR4
RGS1
low risk
high risk
B
risk score
High
Low
p = 0.00029
Survival probability(%)
Time (years)
ROC curve
Sensitivity
AUC at 1 years: 0.57
AUC at 3 years: 0.64
AUC at 5 years: 0.68
1−Specificity
C
Clinical
8−gene signature
Combined
Discrimination
C−index
1−year AUC
3−year AUC
5−year AUC
D
Apparent
Bootstrap−corrected
C−index
1−year AUC
3−year AUC
5−year AUC
Discrimination
Clinical
Combined
E
Median ..C = 0.068
Bootstrap frequency
△C−index (Combined − Clinical)
F
Published signature
Clinical + Published
8-gene signature
Clinical + 8-gene signature
Time−dependent AUC
1−year
3−year
5−year
Time horizon
G
C−index = 0.099 (95% CI 0.038–0.161)
P = 0.002
Harrell's C−index
RGS1 alone
8−gene signature
H
RGS1 alone
8−gene signature
Time−dependent AUC
P = 0.016
P < 0.001
P < 0.001
1−year
3−year
5−year

Figure S3

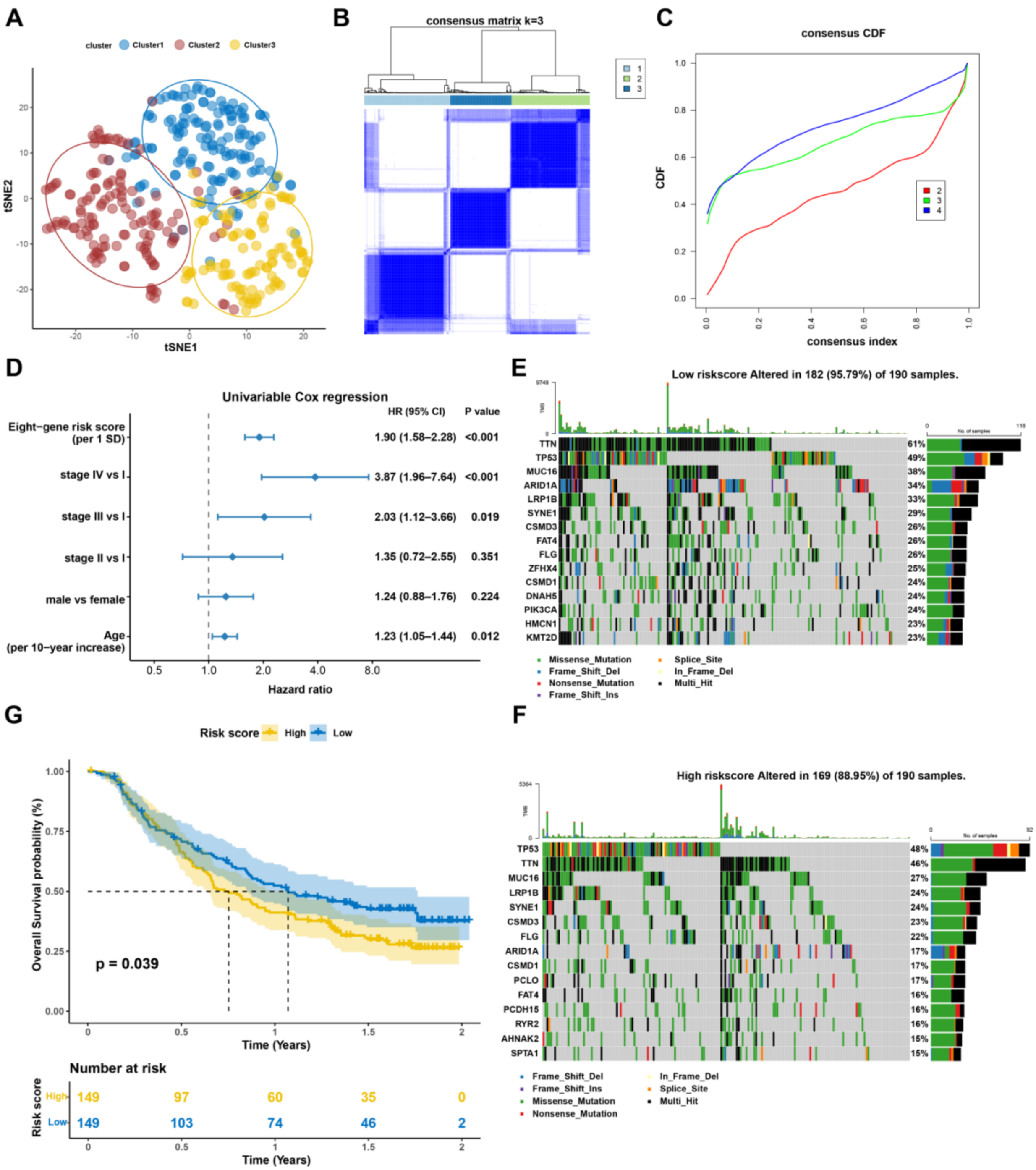
A
cluster Cluster1 Cluster2 Cluster3
tSNE2
tSNE1
B
consensus matrix k=3
1
2
3
C
consensus CDF
CDF
consensus index
2
3
4
D
Univariable Cox regression
HR (95% CI) P value
Eight−gene risk score (per 1 SD) 1.90 (1.58–2.28) <0.001
stage IV vs I 3.87 (1.96–7.64) <0.001
stage III vs I 2.03 (1.12–3.66) 0.019
stage II vs I 1.35 (0.72–2.55) 0.351
male vs female 1.24 (0.88–1.76) 0.224
Age (per 10−year increase) 1.23 (1.05–1.44) 0.012
Hazard ratio
E
Low riskscore Altered in 182 (95.79%) of 190 samples.
TTN 61%
TP53 49%
MUC16 38%
ARID1A 34%
LRP1B 33%
SYNE1 29%
CSMD3 26%
FAT4 26%
FLG 26%
ZFHX4 25%
CSMD1 24%
DNAH5 24%
PIK3CA 24%
HMCN1 23%
KMT2D 23%
Missense_Mutation
Frame_Shift_Del
Nonsense_Mutation
Frame_Shift_Ins
Splice_Site
In_Frame_Del
Multi_Hit
G
Risk score High Low
Overall Survival probability (%)
p = 0.039
Time (Years)
Number at risk
Risk score
High 149 97 60 35 0
Low 149 103 74 46 2
Time (Years)
F
High riskscore Altered in 169 (88.95%) of 190 samples.
TP53 48%
TTN 46%
MUC16 27%
LRP1B 24%
SYNE1 24%
CSMD3 23%
FLG 22%
ARID1A 17%
CSMD1 17%
PCLO 17%
FAT4 16%
PCDH15 16%
RYR2 16%
AHNAK2 15%
SPTA1 15%
Frame_Shift_Del
Frame_Shift_Ins
Missense_Mutation
Nonsense_Mutation
In_Frame_Del
Splice_Site
Multi_Hit

Figure S4

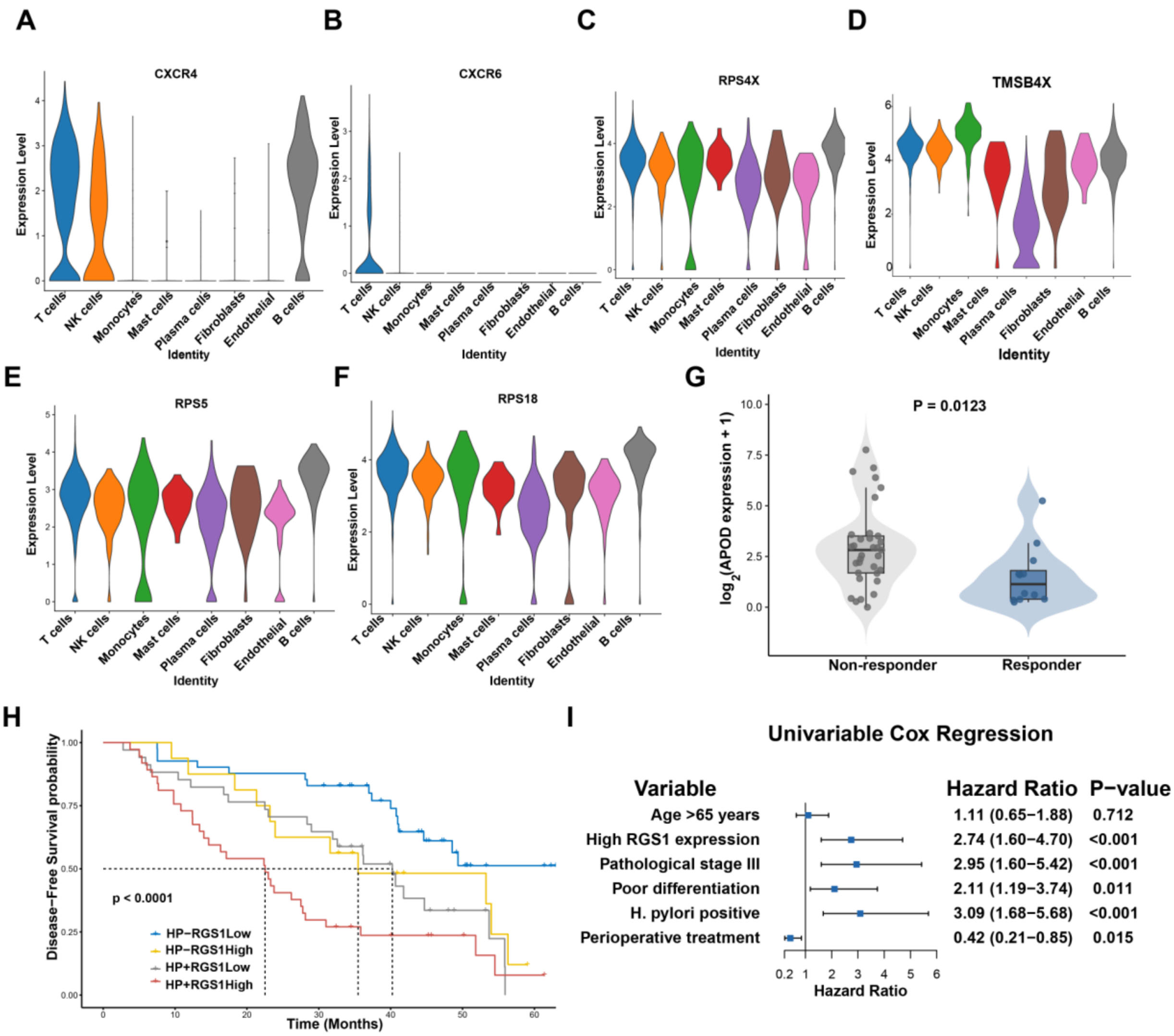
A
CXCR4
B
CXCR6
C
RPS4X
D
TMSB4X
E
RPS5
F
RPS18
G
P = 0.0123
Non-responder
Responder
H
p < 0.0001
HP−RGS1Low
HP−RGS1High
HP+RGS1Low
HP+RGS1High
Disease-Free Survival probability
Time (Months)
I
Univariable Cox Regression
Variable
Hazard Ratio
P−value
Age >65 years
1.11 (0.65−1.88)
0.712
High RGS1 expression
2.74 (1.60−4.70)
<0.001
Pathological stage III
2.95 (1.60−5.42)
<0.001
Poor differentiation
2.11 (1.19−3.74)
0.011
H. pylori positive
3.09 (1.68−5.68)
<0.001
Perioperative treatment
0.42 (0.21−0.85)
0.015
Hazard Ratio
